# An Antiseptic Gargle

**Published:** 1886-10

**Authors:** 


An Antiseptic Gargle.—

| | | |
|---|---|---|
| ℞ Thymol, | - - - - | grs. iv. |
| Acid Benzoic, | - - - - | grs. xlv. |
| Tr. Eucalyptus, | - - - - | ℥ iii. |
| Distilled water, | - - - - | Ojss. M. |

---

The French Academy of Medicine, having been directed by the Government to examine the wines used in France, report in favor of forbidding the sale of all fortified or alcoholized wines. The report also calls for a large reduction of the number of wine shops and for a rigid enforcement of the laws for the suppression of drunkenness.

---

We extend our thanks to Dr. Filippi, of Florence, Italy, editor of *Lo Sperimentale*, for two complete volumes of his journal with the initial number of the present volume. *Lo Sperimentale* is now in its fortieth year and is one of the best medical journals published in any language.

---

Dr. Brown-Sequard has been elected a member of the French Academy of Sciences, after being a candidate for many years. He was elected in the section of medicine and surgery. Among his competitors were Germain-See, Bouchard, Jaccoud, Hayem and Richet.

---

Charrin and Roger have studied the antiseptic action of bile and the bile salts. They find that fresh bile has feeble antiseptic properties. The salts of bile have various antiseptic powers, the taurocholates being the most powerful.

---

Cocaine can now be made artificially. Merck, of Darmstadt, has succeeded in obtaining it from benzoyl-ecgonin, a substance previously discovered by himself.

---

There have now been 103 cases of resection of the pylorus with twenty-nine recoveries. Of these operations Billroth has performed sixteen, with seven recoveries.

---

Aconite Poisoning.—Dr. Ripley of New York considers the hypodermic injection of morphine the most valuable remedy in the treatment of aconite poisoning.

MULTIPLE TÆNIA.—A Russian physician by the administration of male fern, caused the expulsion of 102 tape-worms from one patient.

CASES of hysterical paralysis and aphonia of long standing were rapidly cured by the recent earthquakes at Charleston.

CINCINNATI is to have a crematory, and will use the same style of furnace employed in the Buffalo crematory.

Two female medical students in Paris, one French and the other American, recently fought a duel with swords.

THE State of Georgia has no dissecting law, and body-snatching is a regular trade.

